# Seroepidemiologic studies of hantavirus infection among wild rodents in California.

**DOI:** 10.3201/eid0302.970213

**Published:** 1997

**Authors:** M Jay, M S Ascher, B B Chomel, M Madon, D Sesline, B A Enge, B Hjelle, T G Ksiazek, P E Rollin, P H Kass, K Reilly

**Affiliations:** California Department of Health Services, Sacramento, USA.

## Abstract

A total of 4,626 mammals were serologically tested for antibodies to Sin Nombre virus. All nonrodent species were antibody negative. Among wild rodents, antibody prevalence was 8.5% in murids, 1.4% in heteromyids, and < 0.1% in sciurids. Of 1,921 Peromyscus maniculatus (deer mice), 226 (11.8%) were antibody positive, including one collected in 1975. The highest antibody prevalence (71.4% of 35) was found among P. maniculatus on Santa Cruz Island, off the southern California coast. Prevalence of antibodies among deer mice trapped near sites of human cases (26.8% of 164) was significantly higher than that of mice from other sites (odds ratio = 4.5; 95% confidence interval = 1.7, 11.6). Antibody prevalence increased with rising elevation (> 1,200 meters) and correlated with a spatial cluster of hantavirus pulmonary syndrome cases in the Sierra Nevada.


### Seroepidemiologic Studies of Hantavirus Infection Among Wild Rodents in California

A total of 4,626 mammals were serologically tested for antibodies to Sin Nombre virus. All nonrodent species were antibody negative. Among wild rodents, antibody prevalence was 8.5% in murids, 1.4% in heteromyids, and < 0.1% in sciurids. Of 1,921 *Peromyscus maniculatus* (deer mice), 226 (11.8%) were antibody positive, including one collected in 1975. The highest antibody prevalence (71.4% of 35) was found among *P. maniculatus* on Santa Cruz Island, off the southern California coast. Prevalence of antibodies among deer mice trapped near sites of human cases (26.8% of 164) was significantly higher than that of mice from other sites (odds ratio = 4.5; 95% confidence interval = 1.7, 11.6). Antibody prevalence increased with rising elevation (>1,200 meters) and correlated with a spatial cluster of hantavirus pulmonary syndrome cases in the Sierra Nevada.

In spring 1993, a cluster of unexplained severe acute respiratory illnesses associated with a high death rate was reported in the southwestern United States (1). The outbreak was linked to a newly recognized hantavirus strain, Sin Nombre virus (SNV), carried by the deer mouse, *Peromyscus maniculatus* (2-5). Sporadic cases of the illness, hantavirus pulmonary syndrome (HPS), were subsequently identified in other regions of North America, especially the western United States (6,7). Three additional pathogenic viruses associated with HPS were later discovered outside the usual range of *P. maniculatus*: 1) Black Creek Canal virus, harbored by the cotton rat, *Sigmodon hispidus*, in Florida; 2) Bayou virus, identified in the rice rat, *Oryzomys palustris*, in Louisiana; and 3) New York virus, isolated from the white-footed deer mouse, *Peromyscus leucopus*, in New York (8-12). These viruses have not been found in California; however, two novel hantaviruses, El Moro Canyon virus (EMCV) and Isla Vista virus (ISLA), were recently discovered in that state (13-15). Genetic studies identified the harvest mouse, *Reithrodontomys megalotis*, and the California meadow vole, *Microtus californicus*, as the reservoirs for EMCV and ISLA, respectively. Human infection with EMCV and ISLA has not been documented.

Through 31 January 1997, 156 cases of HPS were reported to the Centers for Disease Control and Prevention (CDC); the case-fatality rate is approximately 50% (T. Ksiazek, P. Rollin, unpub. data). HPS has been confirmed in 26 states, with California reporting the third largest number of cases (14 cases, 8 deaths), after New Mexico (29 cases) and Arizona (22 cases).

Hantaviruses are excreted in the urine, feces, and saliva of asymptomatic infected rodents (16). Transmission to humans occurs when aerosols contaminated with the virus are inhaled (16-18). Bites by infected rodents or exposure to broken skin or mucous membranes may represent alternative routes. Although specific risk factors are poorly defined, persons engaging in activities that bring them in contact with rodents and/or their excretions may be at a higher risk for infection (19).

The present study compiles retrospective and prospective serologic data to 1) confirm *P. maniculatus* as the primary reservoir of SNV and identify alternative reservoirs, if any, in California; 2) assess differences in SNV seroprevalence in vector populations from various geographic regions; and 3) compare seroprevalence rates of reservoirs collected near sites of human cases with those from other sites.

### The Study

### Mammal Surveys

The California Department of Health Services (CDHS) contacted 18 agencies (local vector control districts, local health and environmental health departments, universities, state and national park and forest services, the military, and wildlife refuges) involved in cross-sectional surveys of California mammals to participate in developing a centralized, statewide database of SNV serologic results and ecologic data.

Historical surveys used archived specimens collected in 1975, 1976, and 1988 and stored at the University of California, Berkeley, Museum of Vertebrate Zoology, and specimens collected from 1989 through early 1993 by local vector control districts. Prospective surveys and human case investigations were conducted from late 1993 through the end of 1995. Wild rodent trapping and processing methods were as previously described (20).

Reports by participating agencies included ecologic data on individual mammals (species, age, and sex). Information on survey sites included county, physical location of trapline, date of survey, elevation, and habitat. Habitat was categorized by a single dominant vegetation type: chaparral, conifer trees, grassland, hardwood trees, sage/scrub brush, or urban environment (21).

### Human Cases

Within 2 weeks after a confirmed diagnosis of HPS, the patient or a surrogate was interviewed to determine potential sites of exposure to infected rodents. An environmental/ecologic investigation was conducted at possible sites of exposure (20,22).

### Laboratory Studies

Rodent serum samples were tested by one or more of six laboratories (CDC; CDHS; University of New Mexico; University of California, Davis; and University of Nevada, Reno). Serum samples were examined for IgG antibodies to the SNV nucleocapsid protein by Western blot and/or enzyme-linked immunosorbent assay with CDC reagents (23,24). For archived specimens, liver tissue from frozen carcasses was used for polymerase chain reaction (PCR) (22,25).

### Data Analysis

Descriptive data were analyzed by Epi Info, Version 6.0 (26). Frequency distributions were obtained, and chi-square tests of homogeneity for two-by-two contingency tables were used to examine the statistical significance of any association. A crude relationship between altitude and SNV-antibody prevalence was assessed by the Mantel-Cox test for trend. Adjusted odds ratios and 95% confidence intervals were calculated by logistic-binomial regression (27). A random effects model was chosen because of the presence of important clustering effects from the sampling design. The presence or absence of antibodies to SNV was the primary dependent variable, and characteristics that were identified in the descriptive analysis were the independent variables. Data from samples collected on the Channel Islands were analyzed separately from mainland data where indicated.

### Sin Nombre Virus Antibody Prevalence

### California Mammals

A total of 4,626 (4,549 rodent and 77 nonrodent species) mammals representing 47 species were collected between 1975 and 1995 in California and serologically tested for IgG antibodies to SNV (Table 1). Wild rodents (most 3,109, from the family Muridae) made up 98% of the sample, followed by Sciuridae (1,369), and Heteromyidae (71). Among the Muridae, 78% were deer mice or related species, 11% were wood rats, 6% were domestic rodents (house mice, rats), 3% were harvest mice, and fewer than 1% were meadow voles or cotton rats. Antibodies to SNV were found only among wild rodents. Prevalence of antibodies reactive with SNV was 8.5% among the Muridae, 1.4% among the Heteromyidae, and less than 0.1% among the Sciuridae. Nonrodent species tested included 74 carnivores (five domestic dogs, *Canis familiaris*; 26 coyotes, *Canis latrans*; 25 island foxes, *Urocyon littoralis*; six domestic cats, *Felis domesticus*; two opossums, *Didelphis virginiana*; three striped skunks, *Mephitis mephitis*; seven raccoons, *Procyon lotor*), one black-tailed jackrabbit, *Lepus californicus*, one Nuttall's cottontail, *Silvilagus nuttallii*, and one shrew, *Sorex* sp.

### Deer Mouse Populations

Of 1,921 *P. maniculatus*, 226 (11.8%) had antibodies to SNV (Table 1). Other peromyscine-related species (e.g., cactus mice, canyon mice, California mice, and pinyon mice) also had antibodies to SNV, but prevalence was lower. In almost all instances, infected *P. maniculatus* were collected at the same site as SNV–antibody-positive animals of other species.

Retrospectively, antibodies to SNV were identified in 3 (6.0%) of 50 deer mice specimens collected in 1975 (1 of 22, 4.5%), 1976 (2 of 17, 11.8%), 1981 (0 of 2), 1992 (0 of 1), and early 1993 (0 of 8). The three seropositive *P. maniculatus* collected by the University of California, Berkeley in 1975 and 1976 were from Alameda, Kern, and Mono Counties. Frozen liver tissue available from the seropositive Kern County specimen yielded a PCR sequence of the G1 amplimer of SNV that differed from a *P. maniculatus*

Table 1. Prevalence of antibodies to Sin Nombre virus among wild rodents in California, 1975–1995

| Family/ Species | Common Name | No. Tested | No. Pos. | % Pos. |
|---|---|---|---|---|
| Heteromyidae | | | | |
| *Dipodomys* sp. | kangaroo rats | 28 | 1 | 3.6 |
| *Perognathus* sp. | pocket mice | 43 | 0 | 0.0 |
| SUBTOTAL | | 71 | 1 | 1.4 |
| Muridae | | | | |
| *Microtus* sp. | meadow voles | 29 | 5 | 17.2 |
| *M. californicus* | California meadow vole | 22 | 5 | 22.7 |
| *M. montanus* | Montane vole | 3 | 0 | 0.0 |
| *M. townsendii* | Townsend's vole | 1 | 0 | 0.0 |
| *Mus musculus* | house mouse | 88 | 0 | 0.0 |
| *Neotoma* sp. | wood rats | 330 | 2 | 0.6 |
| *N. cinerea* | bushy-tailed wood rat | 20 | 0 | 0.0 |
| *N. fuscipes* | dusky-footed wood rat | 215 | 2 | 0.9 |
| *N. lepida* | desert wood rat | 95 | 0 | 0.0 |
| *Peromyscus* sp. | deer mice | 2430 | 236 | 9.7 |
| *P. boylii* | brush mouse | 98 | 0 | 0.0 |
| *P. californicus* | California mouse | 159 | 4 | 2.5 |
| *P. crinitus* | canyon mouse | 29 | 2 | 6.9 |
| *P. eremicus* | cactus mouse | 100 | 1 | 1.0 |
| *P. maniculatus* | deer mouse | 1921 | 226 | 11.8 |
| *P. truei* | pinyon mouse | 123 | 7 | 5.7 |
| *Rattus norvegicus* | Norway rat | 11 | 0 | 0.0 |
| *Rattus rattus* | roof rat | 99 | 0 | 0.0 |
| *Reithrodontomys megalotis* | harvest mouse | 108 | 16 | 14.8 |
| *Sigmondon hispidus* | cotton rat | 14 | 0 | 0.0 |
| SUBTOTAL | | 3109 | 263 | 8.5 |
| Sciuridae | | 1369 | 1 | <0.1 |
| *Ammonospermophilus leucurus* | antelope ground squirrel | 4 | 0 | 0.0 |
| *Spermophilus.* sp. | ground squirrels | 1205 | 1 | <0.1 |
| *S. beecheyi* | California ground squirrel | 856 | 1 | 0.1 |
| *Tamias* sp. | chipmunks | 152 | 0 | 0.0 |
| *Tamiascurus douglasii* | Douglas' squirrel | 5 | 0 | 0.0 |
| SUBTOTAL | | 1369 | 1 | <0.1 |
| TOTAL | | 4549 | 245 | 5.4 |

collected in nearby Mono County in 1994 by only five residues out of 274. The largest comparative protein dissimilarity was with a *P. maniculatus* from the Channel Islands, which differed by seven amino acid substitutions.

Among the deer mice for which age (1,165 animals, 87% adults) and sex (1,239 animals, 57% male) were available, SNV antibody prevalence was, respectively, 13.5% in adults and 6.7% in juveniles and 13.0% in males and 10.1% in females. Differences in age (chi-square = 5.5) and sex (chi-square = 2.5) were not significant.

### Geographic Distribution

Thirty-four of the state's 58 counties were surveyed (Table 2). Antibodies to SNV were identified in deer mice from 21 (62%) of these counties (Figure 1). Most samples were from coastal counties (56%), followed by foothill/mountainous (36%) and inland/valley (8%) counties. Among these, the prevalence was 14.5% of 684 in the foothills/mountains, 11.6% of 112 on the coast, and 0.7% of 151 in the inland/valley areas.

One hundred and thirty-nine individual sites were surveyed within 34 counties with a mean of four sites per county (Table 2). Antibody prevalence at individual sites was 0.0% to 71.4% (25 of 35 tested on Santa Cruz Island, Santa Barbara County). In the Sierras, the highest antibody prevalence (50.0% of 52) was found in deer mice captured in Truckee, Nevada County, during a human case-patient investigation (Table 3, Case 3). At least one SNV-antibody-positive mouse was detected at each mainland site where 38 or more mice were tested.

### Channel Islands

The Channel Islands, a group of eight islands located south of the Santa Barbara-Los Angeles coast, are 20 km (Anacapa) to 98 km (San Nicolas) from the mainland and 5 km to 45 km from each other. Despite the proximity between them, SNV-antibody prevalence varied significantly between islands. Antibody prevalence in deer mice trapped on the islands (20.9% of 382) was significantly higher than that of deer mice from the mainland (chi-square = 40.9, $p < 0.001$). In addition, Channel Island sequences differed by approximately 17% to 19% from any mainland California sequences (22,25). The highest prevalence was found on Santa Cruz and Santa Rosa Islands, where, respectively, 25 (71.4%) of 35 and 47 (58%) of 81 deer mice were SNV-antibody positive. Antibody prevalence among deer mice on the other islands was 7 (17.9%) of 39 on San Miguel, 1 (14.3%) of 7 on Santa Catalina, and 1 (2.9%) of 34 on San Clemente. However, deer mice sampled on Anacapa (n=37), San Nicolas (n = 91), and Santa Barbara (n = 58) Islands were all SNV-antibody negative.

Table 2. Prevalence of antibodies to Sin Nombre virus among *Peromyscus maniculatus* by county, California, 1975–1995

| County | No. survey sites | No. tested | No. pos. | % pos. |
|---|---|---|---|---|
| COASTAL | | | | |
| Alameda | 2 | 6 | 1 | 16.7 |
| Contra Costa | 1 | 36 | 0 | 0.0 |
| Del Norte | 2 | 19 | 0 | 0.0 |
| Los Angeles | 10(8) | 112(71) | 13(11) | 11.6(15.5) |
| Marin | 2 | 153 | 3 | 2.0 |
| Mendocino | 1 | 13 | 0 | 0.0 |
| Monterey | 2 | 52 | 5 | 9.6 |
| Orange | 8 | 52 | 3 | 5.8 |
| San Diego | 32 | 131 | 7 | 5.3 |
| San Francisco | 1 | 30 | 0 | 0.0 |
| San Luis Obispo | 2 | 4 | 0 | 0.0 |
| San Mateo | 1 | 40 | 8 | 20.0 |
| Santa Barbara | 6(2) | 294(81) | 86(7) | 29.3(8.6) |
| Sonoma | 1 | 7 | 0 | 0.0 |
| Ventura | 5(3) | 137(9) | 0(0) | 0.0(0.0) |
| SUBTOTAL | 76(68) | 1086(704) | 126(45) | 11.6(6.4) |
| INLAND/VALLEY | | | | |
| Imperial | 1 | 2 | 1 | 50.0 |
| Riverside | 10 | 57 | 0 | 0.0 |
| Sacramento | 1 | 36 | 0 | 0.0 |
| San Bernardino | 4 | 49 | 0 | 0.0 |
| San Joaquin | 1 | 7 | 0 | 0.0 |
| SUBTOTAL | 17 | 151 | 1 | 0.7 |
| FOOTHILLS/MOUNTAINS | | | | |
| Butte | 12 | 115 | 14 | 12.2 |
| El Dorado | 1 | 25 | 0 | 0.0 |
| Glenn | 1 | 4 | 0 | 0.0 |
| Inyo | 2 | 3 | 1 | 33.3 |
| Kern | 5 | 66 | 7 | 10.6 |
| Mariposa | 1 | 46 | 7 | 15.2 |
| Mono | 8 | 107 | 17 | 15.9 |
| Nevada | 1 | 52 | 26 | 50.0 |
| Placer | 2 | 29 | 2 | 6.9 |
| Plumas | 4 | 35 | 1 | 2.9 |
| Shasta | 3 | 30 | 4 | 13.3 |
| Siskiyou | 4 | 117 | 12 | 10.3 |
| Tehama | 1 | 35 | 5 | 14.3 |
| Tulare | 1 | 20 | 2 | 10.0 |
| SUBTOTAL | 46 | 684 | 99 | 14.5 |
| TOTAL | 139 | 1921 | 226 | 11.8 |

*Numbers in parentheses exclude the Channel Islands in Los Angeles (Catalina, San Clemente), Santa Barbara (San Miguel, Santa Barbara, Santa Cruz, Santa Rosa), and Ventura (Anacapa, San Nicolas) counties.

### Habitat

A higher SNV-antibody prevalence was observed in the Sierra Nevada, Great Basin, and southern coastal habitats (Figure 1). Likewise, antibody prevalence was higher among deer mice trapped in vegetation associated with these environments: 15.1% of 531 in conifer, 14.8% of 597 in grassland, 13.4% of 86 in hardwood, 11.2% of 165 in sage/scrub brush, and 5.8% of 474 in chaparral. In urban environments, only 2.9% of 68 deer mice were SNV-antibody positive.

Antibody prevalence among deer mice increased significantly ($p < 0.001$) with rising altitude (Figure 2). In the regression model, adjusted odds ratios steadily increased with elevation, peaking at 4.3 in the 1,800- to 2,100-meter range (95% confidence intervals = 1.3, 16.7) (Table 4). The increased prevalence of SNV antibodies in deer mice trapped at higher elevations correlated with an increased incidence of HPS cases at elevations above 1,200 meters (Table 3).

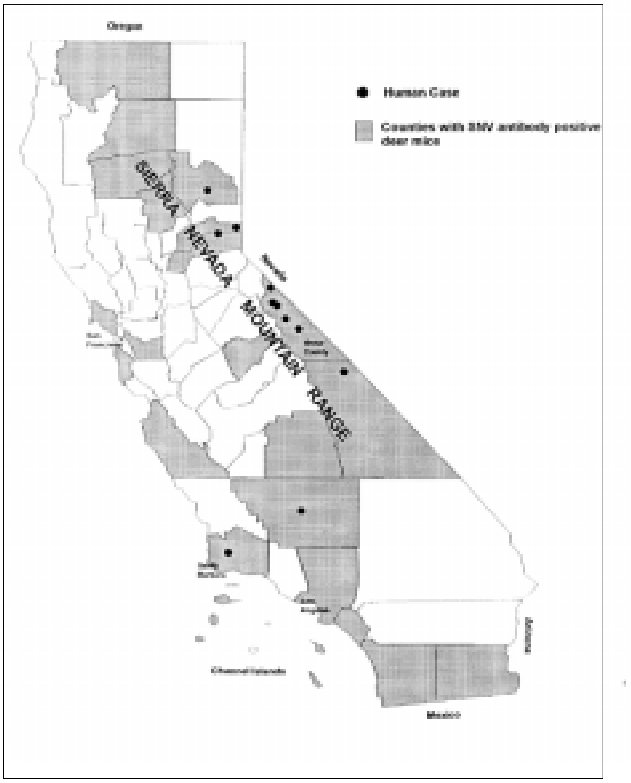

Figure 1. Geographic distribution of hantavirus pulmonary syndrome cases and occurrence of Sin Nombre virus antibodies among deer mice (*Peromyscus maniculatus*), California, 1975–1995 (n=1,921).

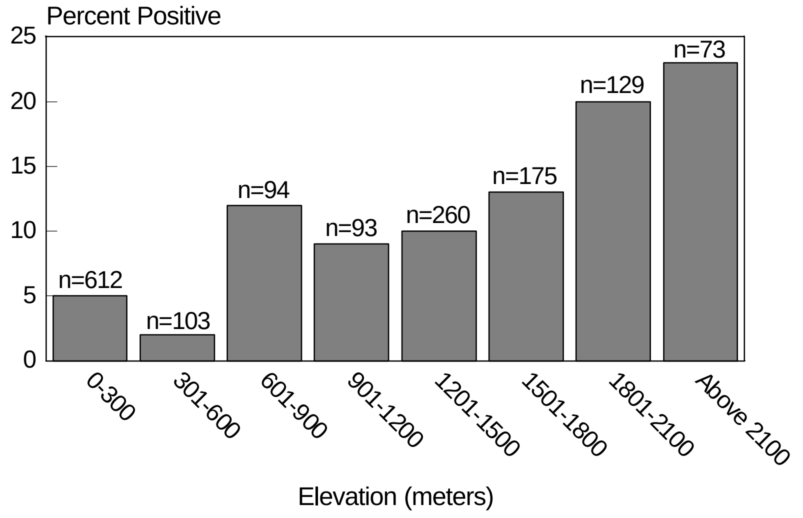

Figure 2. Prevalence of Sin Nombre virus antibodies among deer mice (*Peromyscus maniculatus*) by elevation, California, 1975–1995 (n=1,539, excluding Channel Islands).

### Human Cases

HPS cases were spatially clustered in the state's Sierra Nevada region (Figure 1). Results from antibody prevalence studies among rodents collected during the investigation of six HPS cases in California are presented in Table 3. Antibody prevalence in deer mice trapped at these sites was significantly higher than at sites not associated with a human case (odds ratio = 4.5; 95% confidence interval = 1.7, 11.6) (Table 4).

### Reservoirs of Hantavirus in California

Extensive serologic testing unequivocally identified *P. maniculatus* as the primary reservoir of SNV in California, as did previous studies in the western United States (2,20,28-30). The overall prevalence of SNV antibodies in deer mice (11.9%) in California is also comparable with results published by nearby western states such as Montana (8.0%) and Nevada (12.5%) (28,30). Other peromyscine rodents (e.g., canyon mice and pinyon mice) may harbor the virus in California, but at much lower levels. Because these related wild mice species frequently inhabit the same environment as *P. maniculatus*, their infections may repre-sent transmission from the primary reservoir population rather than from each other, as would occur in a permanent virus-species relationship. The extremely low prevalence of SNV antibodies (Table 1) in heteromyids (kangaroo rats, pocket mice), sciurids (ground squirrels, chipmunks), and Old World murids (domestic mice and rats), despite a large sample size (> 1,600), suggests that these animals are not important in the epidemiology of SNV in California.

Positive serologic test results from California meadow voles and harvest mice probably represent cross-reaction with SNV antigen by ISLA and EMCV, respectively (13-15). The high prevalence of antibodies identified in meadow voles (22.7% of 29) and harvest mice (14.8% of 108) is a cause for concern, even though human infection by these viruses has not been documented. Precautionary measures should be taken against exposure to hantaviruses through all potential

Table 3. Characteristics of selected hantavirus pulmonary syndrome cases and prevalence of antibodies to Sin Nombre virus in *Peromyscus maniculatus* collected at candidate sites of exposure, California, 1994-1995*

| Patient | Onset Date | Outcome | Suspect County of Exposure | Predominant Vegetation | Elevation (meters) | No. Rodents Tested | % Pos. |
|---|---|---|---|---|---|---|---|
| 1 | 09/94 | Nonfatal | Mono | conifer, sage brush | 1801-2100 | 34 | 14.7 |
| 2 | 03/95 | Nonfatal | Mono | conifer, sage brush | 1801-2100 | 22 | 13.6 |
| 3 | 04/95 | Nonfatal | Nevada | sage brush | 1801-2100 | 52 | 50.0 |
| 4 | 06/95 | Fatal | Mono | conifer, sage brush | 1801-2100 | 11 | 54.5 |
| 5 | 09/95 | Fatal | Placer/Nevada | conifer | 1501-1800 | 26 | 7.7 |
| 6 | 10/95 | Nonfatal | Plumas | conifer | 1201-1500 | 19 | 5.3 |
| Total | | | | | | 164 | 26.8 |

*Rodent studies associated with California HPS cases described in greater detail (20,22,29).

wild rodent reservoirs until more is known about these newly discovered strains.

### Temporal and Spatial Trends in Deer Mice Populations

Our data from historical surveys indicate that SNV was already circulating in deer mice 20 years ago in parts of California. The antibody-positive deer mouse originally trapped in Kern County in 1975 is the oldest documented evidence of SNV infection in wild rodents. Notably, SNV-antibody-positive deer mice identified retrospectively were captured almost 20 years before the first HPS cases were recognized in California and in some of the same geographic regions (20,22,29,31). The slight difference (approximately 2%) between sequences from the Kern County deer mouse trapped in 1976 and a Mono County deer mouse trapped in 1994 indicates a long-standing stability of the virus, as previously demonstrated by Nerukar et al. in Mono County (32).

Although SNV infection in deer mice is widespread in California, represented biotypes vary considerably in seroprevalence levels. For example, deer mice in foothill/mountainous (14.5% of 684) counties have a higher seroprevalence than those in inland/valley (0.7% of 151) and coastal (6.4% of 704, excluding the Channel Islands) counties. The trend of increasing prevalence with rising elevation found in this analysis has been observed in other states (Jim Mills, pers. comm.).

The Channel Islands offered a unique opportunity to study SNV infection in a relatively isolated population of deer mice. Deer mouse populations have been on the Channel Islands long enough that each island has its own subspecies and have considerable variation genetically within those subspecies (33,34). Likewise, Hjelle et al. found significant divergence between genetic sequences of virus from infected deer mice collected on the islands and those from the nearby coastal mainland (25). Although travel from the mainland to the islands and from island to island is common, evidence suggests that SNV coevolved separately within the deer mouse populations on each island (San Clemente, San Miguel, Santa Catalina, Santa Cruz, Santa Rosa). It appears that the virus is not endemic or is present at very low levels among deer mice on Anacapa, San Nicolas, and Santa Barbara Islands.

### Human Cases

The antibody prevalence of SNV in deer mice collected at potential exposure sites during the investigation of sporadic HPS cases in California (26.8%) was similar to the antibody prevalence (30.0%) in deer mice observed during the 1993 HPS outbreak in the Four Corners region (2). Prevalence was significantly higher ($p = 0.002$) in deer mice trapped near human case exposure sites than in those from survey sites with no cases (Table 4). Together these findings imply that the percentage of infected deer mice may be a risk factor for human exposure to SNV. In addition, landscape features such as high elevation may be another important predictor of hantavirus in the state. The spatial clustering of HPS cases in the Sierra Nevada range supports this conclusion.

Characteristics of the mouse population, the environment, or human lifestyles may explain these geographic differences. The climate and vegetation in the mountains could be conducive to large populations of deer mice, a factor which might influence SNV prevalence. In addition, the occupational and recreational activities of the inhabitants of rural, mountainous environments bring them into frequent contact with rodents. In addition, local residences (e.g., old log cabins) are prone to rodent infestation, a possible risk factor for HPS infection (35).

Otteson et al. documented a higher SNV antibody prevalence among rodents trapped near

Table 4. Odds ratios for Sin Nombre virus antibody prevalence among *Peromyscus maniculatus* by association with human cases and elevation, California, 1975–1995[a]

| | Cases (+SNV) | Controls (-SNV) | Adj. OR[bc] | 95% CI[c] | p value |
|---|---|---|---|---|---|
| Human case[d] | | | | | |
| Absent | 101 | 1274 | 1.0 | NA[c] | NA |
| Present | 44 | 120 | 4.5 | 1.7-11.6 | 0.002 |
| Elevation (meters) | | | | | |
| 0-300 | 32 | 580 | 1.0 | NA | NA |
| 301-600 | 2 | 101 | 0.4 | 0.4-3.5 | 0.423 |
| 601-900 | 11 | 83 | 0.6 | 0.2-1.8 | 0.390 |
| 901-1200 | 8 | 85 | 2.4 | 0.7-8.6 | 0.168 |
| 1201-1500 | 26 | 234 | 3.2 | 1.0-10.0 | 0.044 |
| 1501-1800 | 23 | 152 | 2.6 | 0.7-9.2 | 0.151 |
| 1801-2100 | 26 | 103 | 4.3 | 1.3-16.7 | 0.032 |

[a]Excludes Channel Islands; [b]Adjusted for trapline location and survey date, vegetation, sex and age of deer mouse; [c]OR, odds ratio; CI, confidence interval; NA, not applicable; [d]Deer mice collected at candidate sites of exposure during human case investigations.

buildings, regardless of the presence of a human case; a case-control study of the Four Corners outbreak had similar findings (30,36). Information on proximity to human dwellings was not available for our analysis; however, most mice were collected near buildings during investigation of human cases. Since other survey sites were probably less likely to be located near human dwellings, this factor may represent a source of bias in our study. Other biases may have been introduced because of nonrandom sampling and small sample size at some survey sites. Systematic longitudinal studies of SNV infection in deer mice at the key locations identified in this analysis are needed to further develop a predictive model for hantavirus infection in California which could elucidate the natural history of SNV and enhance prevention efforts.

### Public Health Implications

Preliminary results indicate a need for health education of residents, visitors, and workers at high risk, especially in the Sierra Nevada range. Human dwellings in the mountains may be more vulnerable to deer mice infestation, especially if the buildings are older and/or intermittently occupied. In addition, persons working in or cleaning these structures may be at an even higher risk (22,29,35,36). Local health care providers and tertiary care centers should be aware of the potential for HPS cases in the state.

### Acknowledgments

The authors thank the many workers who conducted field surveys and participated in the compilation of a state-wide mammal database; those providing laboratory support, including David Cottam, Dale Dondero, CDHS; Mary Lane Martin, George Gallucci, and Brad Hotard, CDC; Jeff Riolo, University of Nevada, Reno; and Vicki Kramer for her helpful comments and review of this manuscript. This work was supported in part by Public Health Service Grant RO1 AI36336.

**Michele Jay,* Michael S. Ascher,† Bruno B. Chomel,‡ Minoo Madon,§ David Sesline,† Barryett A. Enge,† Brian Hjelle,¶ Thomas G. Ksiazek,¥ Pierre E. Rollin,¥ Philip H. Kass,‡ and Kevin Reilly***

*California Department of Health Services, Sacramento, California, USA; †Viral and Rickettsial Diseases Laboratory, Berkeley, California, USA; ‡University of California, Davis, California, USA; §California Department of Health Services, Ontario, California, USA; ¶University of New Mexico, Albuquerque, New Mexico, USA; ¥Centers for Disease Control and Prevention, Atlanta, Georgia, USA

### References

1. Centers for Disease Control and Prevention. Outbreak of acute illness - southwestern United States, 1993. MMWR Morb Mortal Wkly Rep 1993;42:421-4.
2. Childs JE, Ksiazek TG, Spiropoulou CF, Krebs JW, Morzunov S, Maupin GO, et al. Serologic and genetic identification of *Peromyscus maniculatus* as the primary rodent reservoir for a new hantavirus in the Southwestern United States. J Infect Dis 1994;169:1271-80.
3. Hjelle B, Jenison S, Torrez-Martinez N, Yamada T, Nolte K, Zumwalt R, et al. A novel hantavirus associated with an outbreak of fatal respiratory disease in the southwestern United States: evolutionary relationships to known hantaviruses. J Virol 1994;68:592-6.
4. Elliott LH, Ksiazek TG, Rollin PE, Spiropoulou CF, Morzunov S, Monroe M, et al. Isolation of the causative agent of hantavirus pulmonary syndrome. Am J Trop Med Hyg 1994;51:102-8.
5. Nichol ST, Spiropoulou CF, Morzunov S, Rollin PE, Ksiazek TG, Feldmann H, et al. Genetic identification of a hantavirus associated with an outbreak of acute respiratory illness. Science 1993;262:914-7.
6. Khan AS, Khabbaz RF, Armstrong LR, Holman RC, Bauer SP, Graber J, et al. Hantavirus pulmonary syndrome: the first 100 cases. J Infect Dis 1996;173:1297-303.
7. Centers for Disease Control and Prevention. Hantavirus pulmonary syndrome - United States, 1995 and 1996. MMWR Morb Mortal Wkly Rep 1996;45:291-5.
8. Morzunov SP, Feldmann H, Spiropoulou CF, Semenova VA, Rollin PE, Ksiazek TG, et al. A newly recognized virus associated with a fatal case of hantavirus pulmonary syndrome in Louisiana. J Virol 1995;69:1980-3.
9. Rollin PE, Ksiazek TG, Elliott LH, Ravkov EV, Martin ML, Morzunov S, et al. Isolation of Black Creek Canal virus, a new hantavirus from *Sigmondon hispidus* in Florida. J Med Virol 1995;46:35-9.
10. Hjelle B, Lee SW, Song W, Torrez-Martinez N, Song JW, Yanagihara R, et al. Molecular linkage of hantavirus pulmonary syndrome to the white-footed mouse, *Peromyscus leucopus*: genetic characterization of the M genome of New York virus. J Virol 1995;69:8137-41.
11. Torrez-Martinez N, Hjelle B. Enzootic of Bayou hantavirus in rice rats (*Oryzomys palustris*) in 1983. Lancet 1995;346:780-1.
12. Song J, Baek LJ, Gajdusek DC, Yanagihara R, Gavrilovskaya I, Luft BJ, et al. Isolation of pathogenic hantavirus from the white footed mouse (*Peromyscus leucopus*). Lancet 1994;344:1637.
13. Hjelle B, Chavez-Giles F, Torrez-Martinez N, Yates T, Sarisky J, Webb J, et al. Genetic identification of a novel hantavirus of the harvest mouse *Reithrodontomys megalotis*. J Virol 1994;68:6751-4.
14. Song W, Torrez-Martinez N, Irwin W, Harrison FJ, Davis R, Ascher M, et al. Isla Vista virus: a genetically novel hantavirus of the California vole *Microtus californicus*. J Gen Virol 1995;76:3195-9.
15. Rowe JE, St. Jeor SC, Riolo J, Otteson EW, Monroe MC, Henderson WW, et al. Coexistence of several novel hantaviruses in rodents indigenous to North America. Virology 1995;213:122-30.
16. LeDuc JW. Epidemiology of Hantaan and related viruses. Lab Anim Sci 1987;37:413-8.

17. Tsai TF. Hemorrhagic fever with renal syndrome: mode of transmission to humans. Lab Anim Sci 1987;37:428-30.
18. Xu ZY, Guo CS, Wu YL, Zhang XW, Liu K. Epidemiological studies of hemorrhagic fever with renal syndrome: analysis of risk factors and mode of transmission. J Infect Dis 1985;152:137-44.
19. Centers for Disease Control and Prevention. Hantavirus infection - southwestern United States: interim recommendations for risk reduction. MMWR Morb Mortal Wkly Rep 1993;42(RR-11):ii-13.
20. Turell MJ, Korch GW, Rossi CA, Sesline D, Enge BA, Dondero DV, et al. Short report: prevalence of hantavirus infection in rodents associated with two fatal human infections in California. Am J Trop Med Hyg 1995;52:180-2.
21. Ornduff R. Introduction to California plant life. Berkeley and Los Angeles: University of California Press, 1974:152.
22. Hjelle B, Torrez-Martinez N, Koster FT, Jay M, Ascher MS, Brown T, et al. Epidemiologic linkage of rodent and human hantavirus genomic sequences in case investigations of hantavirus pulmonary syndrome. J Infect Dis 1996;173:781-6.
23. Yamada T, Hjelle B, Lanzi R, Morris C, Anderson B, Jenison S. Antibody responses to Four Corners hantavirus infections in the deer mouse (*Peromyscus maniculatus*): identification of an immunodominant region of the viral nucleocapsid protein. J Virol 1995;69:1939-43.
24. Feldmann H, Sanchez A, Morzunov S, Spiropoulou CF, Rollin PE, Ksiazek TG, et al. Utilization of autopsy RNA for the synthesis of the nucleocapsid antigen of a newly recognized virus associated with hantavirus pulmonary syndrome. Virus Res 1993;30:351-67.
25. Hjelle B, Chavez-Giles F, Torrez-Martinez N, Yamada T, Sarisky J, Ascher M, et al. Dominant glycoprotein epitope of four corners hantavirus is conserved across a wide geographical area. J Gen Virol 1994;75:2881-8.
26. Dean JA, Coulombier D, Smith DC, Brendel KA, Arner TG, Dean AG. Epi Info, version 6: a word processing, database, and statistics program for public health on IBM-compatible microcomputers. Atlanta: Centers for Disease Control and Prevention, 1994:601.
27. EGRET. Seattle: Statistics and Epidemiological Research Corporation, 1995.
28. Douglass RJ, Van Horn R, Coffin KW, Zanto SN. Hantavirus in Montana deer mouse populations: preliminary results. J Wildl Dis 1996;32:527-30.
29. Jay M, Hjelle B, Davis R, Ascher M, Baylies HN, Reilly K, et al. Occupational exposure leading to hantavirus pulmonary syndrome in a utility company employee. Clin Infect Dis 1996;22:841-4.
30. Otteson EW, Riolo J, Rowe JE, Nichol ST, Ksiazek TG, Rollin PE, et al. Occurrence of hantavirus within the rodent population of northeastern California and Nevada. Am J Trop Med Hyg 1996;54:127-33.
31. Shefer AM, Tappero JW, Bresee JS, Peters CJ, Ascher MS, Zaki SR, et al. Hantavirus pulmonary syndrome in California: report of two cases and investigation. Clin Infect Dis 1994;19:1105-9.
32. Nerukar VR, Song JW, Song KJ, Nagle JW, Hjelle B, Jenison S, et al. Genetic evidence for a hantavirus enzootic in deer mice (*Peromyscus maniculatus*) captured a decade before the recognition of hantavirus pulmonary syndrome. Virology 1994;204:563-8.
33. von Bloeker JC. Land mammals of the southern California islands. Santa Barbara (CA): Santa Barbara Botanic Garden, 1967:106.
34. Gill AE. Evolutionary genetics of California Islands *Peromyscus*. In: Power DM, editor. The California Islands. Santa Barbara (CA): Santa Barbara Museum of Natural History, 1980:719-43.
35. Armstrong LR, Zaki SR, Goldoft MJ, Todd RL, Khan AS, Khabbaz RF, et al. Hantavirus pulmonary syndrome associated with entering or cleaning rarely used, rodent-infested structures. J Infect Dis 1995;172:1166.
36. Childs JE, Krebs JW, Ksiazek TG, Maupin GO, Gage KL, Rollin PE, et al. A household-based, case-control study of environmental factors associated with hantavirus pulmonary syndrome in the southwestern United States. Am J Trop Med Hyg 1995;52:393-7.
